# Profiles of children’s physical activity and sedentary behaviour between age 6 and 9: a latent profile and transition analysis

**DOI:** 10.1186/s12966-018-0735-8

**Published:** 2018-10-23

**Authors:** Russell Jago, Ruth Salway, Deborah A. Lawlor, Lydia Emm-Collison, Jon Heron, Janice L. Thompson, Simon J. Sebire

**Affiliations:** 1 Centre for Exercise, Nutrition & Health Sciences, School for Policy Studies, University of Bristol, 8 Priory Road, Bristol, BS8 1TZ UK; 2 MRC Integrative Epidemiology Unit at the University of Bristol, Oakfield House, Oakfield Grove, Bristol, BS8 2BN UK; 3 Population Health Sciences, Bristol Medical School, University of Bristol, Canynge Hall, Whiteladies Road, Bristol, BS8 2PS UK; 4 School of Sport, Exercise and Rehabilitation Sciences, University of Birmingham, Birmingham, B15 2TT UK

**Keywords:** Physical activity, Profile, Transition, Cohort, Children, Weekend

## Abstract

**Background:**

Physical activity is associated with improved physical and mental health among children. However, physical activity declines and sedentary time increases with age, and large proportions of older children do not meet the recommended hour per day of moderate-to-vigorous-intensity physical activity (MVPA). The aim of this paper is to identify profiles of children based on the complex relationship between physical activity and sedentary time at ages 6 and 9 and explore how those profiles are associated with other covariates and how they change over time.

**Methods:**

Valid accelerometer data were collected for 1132 children aged 6 and 1121 at age 9, with 565 children with data at both ages. We calculated the proportions of total wear time spent in sedentary, light and MVPA activity on both weekdays and weekends. Latent profile (class) analysis was applied separately to the two age groups to identify activity profiles. We then used latent transition analysis to explore transitions between profiles at the two time points.

**Results:**

We identified five profiles of activity at age 6 and six profiles at age 9. Although profiles were not directly equivalent, five classes captured similar patterns at both ages and ranged from very active to inactive. At both ages, active profiles, where the majority achieved the recommended MVPA guidelines, were more likely to be active at weekends than on weekdays. There was substantial movement between classes, with strongest patterns of movement to classes with no change or a decrease in MVPA. Transition between classes was associated with sex, BMI z-score, screen-viewing and participation in out-of-school activities.

**Conclusions:**

This paper is the first to apply latent profile analysis to the physical activity of UK children as they move through primary school. Profiles were identified at ages 6 and 9, reflecting different weekday and weekend patterns of physical activity and sedentary time. There was substantial movement between profiles between ages 6 and 9, mostly to no change or less active profiles. Weekend differences suggest that greater focus on how weekend activity contributes to an average of 60 min per day of MVPA across the week may be warranted.

**Electronic supplementary material:**

The online version of this article (10.1186/s12966-018-0735-8) contains supplementary material, which is available to authorized users.

## Background

Physical activity is associated with improved physical and mental wellbeing among children and young people [1]. There is also some evidence that sedentary behaviour is associated with adverse health outcomes among children and young people but there is currently some debate as to whether these associations are independent of physical activity [2, 3]. The amount of physical activity in which children engage declines as they move through childhood and into adulthood, with large proportions of older children and adolescents not engaging in the recommended hour per day of moderate-to-vigorous-intensity physical activity (MVPA) [4, 5]. Conversely, large-scale international studies have shown that sedentary behaviour increases as children age [4]. Increasing physical activity [6] and reducing sedentary time [7] are both issues of global importance but attempts to increase children’s physical activity and reduce sedentary time have had limited impact, suggesting that new ways of helping children to be more active and less sedentary are required [8].

The relationship between physical activity and sedentary behaviour is complex [9]. While sedentary behaviour is often defined (academically) as not simply a state of physical inactivity, but a separate and distinct behaviour, parents often view reducing sedentary behaviour as key to increasing their child’s physical activity and vice versa [10]. Several international bodies have suggested that movement behaviours should be considered that integrate physical activity and sedentary time across the day rather than focusing on just one behaviour [11, 12]. Furthermore, several studies have suggested that re-allocating sedentary time to light intensity physical activity would elicit reductions in the risk factor profile of children and adults [13, 14]. Thus, there is a need to consider overall movement behaviour profiles of children to identify if there are patterns of behaviour that: a) may be associated with reduced health risk; b) show a less steep decline in overall physical activity from childhood to adolescence; and c) offer insights into how to help children reach and maintain sufficient levels of activity to promote health.

Cluster analysis [15] and latent class analysis [16] are methods used to identify groups of people who share similar characteristics. These methods are especially useful when characteristics combine in complex ways and therefore applying these to children’s physical activity and sedentary behaviours could help to increase our understanding of these behaviours. Evidence-based cluster analysis may define groups based on health-related cut-points, whereas more data-driven methods use the data themselves to determine appropriate clusters. Unlike cluster analysis, latent class analysis uses an underlying probabilistic model which means that the uncertainty in class membership can be estimated and included in standard statistical techniques [16]. Traditionally, latent class analysis (LCA) refers to the situation where these variables are categorical, and latent profile analysis (LPA) when they are continuous although, in practice, there is not such a clear distinction and it is possible to combine categorical and continuous variables [17]. Latent class analysis has been used in other public health applications such as substance use, smoking, exercise and dietary behaviour [18–21], but has been less common in studies of physical activity, especially among children. A recent review [22] found that the majority of studies in the physical activity context used cluster analysis. These cluster analyses were mainly cross-sectional studies involving older children (> 9 years) or adolescents, and consistently found girls identified within clusters characterised by low physical activity. Among studies applying LCA, one cross-sectional study used objective accelerometer data to measure physical activity in children under 11 [23], and MVPA and sedentary time were analysed separately thus not including interactions between different types of activity. To date, longitudinal studies have used the same fixed classes at different time points, and thus not considered how classes might have changed over time. A large cohort analysis [24] using self-report physical activity data in adolescents aged 11–21 reported overall changes in the distribution of classes but did not look at movement between classes. A much smaller study [25] used accelerometer data in children 5–6 and 10–12 and reported change between profiles, but sample sizes were small and did not allow any investigation of factors associated with transitions. There are no studies in younger children using accelerometer-measured MVPA and sedentary time that look at whether the activity profiles change over time or seek to identify factors that are associated with movement between profiles.

The aim of this paper is to identify profiles of children based on the complex relationship between physical activity and sedentary time at ages 6 and 9 and explore how those profiles may change over time and how children move between them. In addition, we aim to explore how these profiles and transitions are associated with factors such as sex, BMI, deprivation and activities.

## Methods

Data are from the B-PROACT1V study, a longitudinal study that aimed to examine the physical activity and sedentary behaviours of primary school children aged 5–11 years, and their parents [5, 26, 27]. The study received ethical approval from the School of Policy Studies Ethics Committee at the University of Bristol, UK, and written parental consent was received for all participants [28]. In Phase 1, all children in Year 1 of primary school (aged 5–6 years) from 57 schools in and around Bristol were invited to participate, with data collection taking place between January 2012 and July 2013. In Phase 2, when the children were in Year 4 (aged 8–9 years), all schools from Phase 1 were invited to participate, with 47 schools agreeing. All children were eligible to participate regardless of whether they had participated in Phase 1, and data collection took place between March 2015 and July 2016. Data were collected for 1299 children in Year 1 and 1223 children in Year 4, with 685 children included in both phases.

### Accelerometer data

Children wore a waist-worn ActiGraph wGT3X-BT accelerometer for 5 days, including two weekend days. Accelerometer data were processed using Kinesoft (v3.3.75; Kinesoft, Saskatchewan, Canada) and analysis was restricted to those children who provided at least 2 days of valid weekday data and one valid weekend day to provide a compromise between a typical day and maximising the sample size. A valid day was defined as at least 500 min of data, after excluding intervals of ≥60 min of zero counts allowing up to 2 min of interruptions [4]. Valid data were available for 1132 children at age 6, 1121 at age 9 and 565 at both ages. Data were recorded at 10 s intervals and characterised as sedentary, light or MVPA using Evenson population-specific cut points for children [29]. The average number of MVPA and sedentary minutes per day were derived for each child, and average minutes for weekday and weekend were calculated. Wear times differed between children, depending on the time of year of data collection and between ages 6 and 9. To avoid wear-time related bias in the latent classes, we used the proportion of total wear time spent in sedentary, light and MVPA activity.

### Other measurements

Child height and weight were measured, and body mass index (BMI) was calculated and converted to an age- and sex-specific standard deviation score based on UK reference curves [30, 31]. Indices of Multiple Deprivation (IMD) scores, based on the English Indices of Deprivation (http://data.gov.uk/dataset/index-of-multiple-deprivation), were assigned to each child based on their reported home postcode. Higher IMD scores indicate a greater level of deprivation.

To understand the contribution of specific domains of physical activity and sedentary behaviour to different physical activity profiles, further details of screen-viewing and activity were obtained. In both years, parents were asked about the number of hours their child typically spent in various screen-viewing activities on weekdays and at weekends (e.g. TV, tablets & games consoles, coded from 0 = ‘None’ to 5 = ‘4 h or more’) and these were combined to give a total number of average hours spent in screen-viewing on weekdays and weekends. Children completed a short questionnaire, in which they were asked about the frequency (coded from 0 = ‘Never’ to 3 = ‘5 days per week’) with which they engaged in different forms of activity outside school hours: sport or exercise club at school, sport or exercise club elsewhere, playing outdoors in their neighbourhood, and playing outdoors at home [32]. These were combined to form three variables: a score 0–6 representing participation in structured activity (clubs), a score 0–6 representing unstructured activity (playing) and a total activity participation score from 0 to 12. In all cases, a higher value indicates a higher frequency of participation in activities outside school. This activity participation variable provides information on the type of activity rather than intensity, and has been shown to be an important predictor of activity with patterns that differ between girls and boys [32].

### Statistical analysis

#### Latent profile and latent transition analysis

Latent class analysis is a latent variable model that can be used to identify underlying homogenous subgroups in a population, based on one or more observed variables which may be categorical or continuous; in the latter case the analysis is often called latent profile analysis [17] or finite mixture modelling [33]. Individuals are assumed to belong to one of a set of mutually exclusive latent (unobserved) classes, and in latent profile analysis the observed variables are assumed to be normally-distributed within these classes. As the underlying model is probabilistic, latent profile analysis estimates the parameters of the within-class distributions, and probabilities of class membership for participants. This probabilistic assignment of participants into classes enables uncertainty in class-membership to be appropriately modelled and represents a benefit of the latent approach over methods such as cluster analysis. Latent class analysis allows participants to be included in analyses as long as they have one measure (i.e. they can have partial missing data) through the use of Maximum Likelihood estimation under the assumption that data are missing at random.

Latent transition analysis [16] is a longitudinal extension of latent class/profile analysis requiring the estimation of a latent class model at two or more time-points. Transitions between these time points are modelled via a transition matrix that describes the movement between states through time. With a standard latent class analysis, the focus is typically on the use of explanatory variables to predict class membership (handled via a multinomial regression model). With latent transition analysis, both class membership (time 1 and time 2) and also the transition probabilities may depend on explanatory variables (for instance, a baseline predictor may increase the probability of a participants moving from a high- to low-activity state). All analysis was performed using Mplus v8 [34].

#### Cross-sectional models

We fitted cross-sectional latent profile analysis models for age 6 and 9 separately, using as class variables the proportions of time spent in MVPA and sedentary time for weekdays and weekends (light activity is implicitly included as all three proportions must sum to one). We assumed that the weekday and weekend sedentary proportion variances were the same within a class (and likewise for the MVPA variances) but allowed sedentary variances to differ from the MVPA variances, and both to differ across classes. Latent profile models usually assume conditional independence which is theoretically violated here as the proportions are correlated. To account for this, we allowed a residual covariance between weekday sedentary and MVPA proportions and the same for weekend proportions. We explored a few alternative specifications to assess the sensitivity of the final models to these assumptions, especially concerning the variance.

There is no single commonly-accepted criterion to determine the number of classes [35], so we used a mixture of statistical criteria, interpretability and parsimony. We initially fit latent profile models for 2–10 classes, and reported the Bayesian Information Criterion [36] (BIC), where a lower value indicated better model fit, and the Lo-Mendell-Rubin (LMR) [37] and bootstrapped likelihood ratio (BLRT) tests [33]. These tests both compare a k-1 versus a k-class model, with a low *p*-value rejecting the k-1 class model in favour of the k class model. These criteria have been shown to perform well at identifying an appropriate number of classes [35, 38]. We also reported the relative entropy, an overall measure of classification on a scale of 0 (random) to 1 (perfect classification) [39] and the smallest class size, to identify problematic models with very small class sizes. We considered the interpretability of the final classes in terms of physical activity behaviour and chose a smaller number of classes when all other considerations are equal. Latent variable models were rerun with multiple start values to ensure that the maximum log-likelihood value was replicated.

Once the number of classes was chosen based on the steps outlined above, we applied descriptive labels to each class based on the estimated profile of sedentary/light/MVPA proportions within each class. Finally, to aid interpretation, we also estimated the expected proportion in each class meeting physical activity guidelines (MVPA> 60 min, based on average wear-time) based on the estimated parameters in each class. Once the classes had been identified we examined whether there were differences between them in terms of sex, standardised BMI z-score, IMD, number of hours of screen viewing and participation in out-of-school activities (for age 9 only) with a Wald test using the BCH method, which includes the classification error and is robust to assumption violations [40].

#### Longitudinal model

A latent transition model [41–43] was used to examine change in class membership for the 565 children who have valid data at both time points. This process combines a cross-sectional estimate of the latent classes at age 6 with a longitudinal description of change over time between ages 6 and 9. Constraining the latent classes to be the same at both time points (measurement invariance) would aid interpretation, so we fit both measurement invariance and non-invariance models to explore whether this assumption is valid. We used a 3-step approach [42, 43] to investigate relationships between the latent classes and explanatory variables. This separates the estimation of the latent classes from the larger structural equation model and ensures that the latent classes do not change when including transitions and/or covariates, whilst still accounting for the measurement error in class assignment. Transition probabilities were modelled as a function of each covariate individually.

#### Missing data

A total of 1690 children with valid accelerometer data at at least one time point were included. The model for age 6 was based on 1087 weekday accelerometer measurements and 980 weekend measurements, and the model for age 9 was based on 1059 weekday and 942 weekend measurements. Missing covariate information (Additional file 1: Table S1) varied from < 1% (z-BMI score at age 9) to 18% (activity participation score at age 9), with a total of 964 participants (80%) at age 6 and 922 cases (79%) at age 9 having complete data, and 449 (66%) with complete data at both time points.

Although there are missing outcome and covariable data in this study it was not possible to use standard multiple imputation techniques [44] as the latent structure of the classes is unknown and as such each imputation could produce different classes. Instead, we used full information maximum likelihood, which uses available information from all participants and handles missing data within the analysis model, assuming that data is missing at random. This has been shown to produce unbiased parameter estimates and standard errors in structural equation models when data are missing at random [45].

## Results

Participant characteristics of the data at ages 6 and 9 are summarised in Additional file 1: Table S1. Average MVPA on weekdays decreased by 5.3 mins (95% CI: 3.4 to 7.2 mins) between age 6 and 9 and average weekday sedentary time increased by 73.2 mins (95% CI: 67.2 to 79.3 mins). There are similar differences in weekend activity with an average decrease of 3.4 mins (95% CI: 0.1 to 6.6 mins) and increase of 64.6 mins (95% CI: 55.3 to 73.9 mins) for MVPA and sedentary time respectively.

### Age 6 cross-sectional latent profiles

Additional file 1: Table S2 reports indicators of model fit for 2–10 classes. The 6-class model had the lowest BIC but identified one very small class (1%) of very high-sedentary low-MVPA outliers. These outliers appear to be heavily influencing the classes in some models, so we excluded them (*n* = 4) in a sensitivity analysis and found a 5-class model fitted best, with class profiles very similar to the remaining 5 classes from the full data. This suggested that the main influence of the outliers was the formation of the extra class. As this class was too small for further analysis and caused identification and interpretation problems in further analyses, we have reported the 5-class model without outliers in the remainder of the paper (*n* = 1128). The full 6-class model that included the outliers is described in Additional file 1: Table S3 for comparison. We explored several alternative model specifications and found that, while the exact number of classes differed, similar types of profile kept arising, providing support for the 5-class model.

#### Latent profiles at age 6

**Fig. 1:**
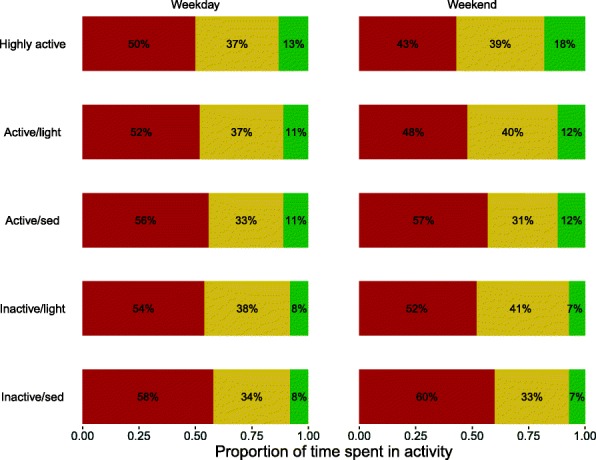
Class profiles for Age 6. Proportions of time spent in sedentary (red), light (yellow) and MVPA (green) at weekdays and weekends for the 5 classes identified at age 6. Classes are ordered roughly from most active to least active

The weekday and weekend profiles for each class are shown in Fig. 1, and the estimated proportions of children in each class are shown in Fig. 3 (left panel; see also Additional file 1: Table S3).

To aid interpretation we have organised the classes approximately in order from most active (highest MVPA) to least active (MVPA). The five classes were identified as follows:

- **Highly active** (9%): High and very high levels of MVPA, especially at weekends, combined with low sedentary proportion. Nearly all meet the recommended level of MVPA of 60+ mins/day (92% on weekdays and 100% on weekends).
- **Active/light** (29%): Higher MVPA than average and average to low sedentary proportion; a large proportion of non-sedentary time is light activity. Most meet the recommended MVPA (77% on weekdays and 87% on weekends).
- **Active/sed** (19%): Higher MVPA than average (similar to *Active/light*) but above average sedentary proportion. Most meet the recommended MVPA (77% and 86%).
- **Inactive/light** (15%): Low MVPA combined with average sedentary proportion; a large proportion of non-sedentary time is light activity. A minority met the recommended MVPA (33% on weekdays and 19% on weekends)
- **Inactive/sed** (28%): Low MVPA (similar to *Inactive/Light*) but combined with high sedentary proportion. A minority met the recommended MVPA (33% on weekdays and 20% on weekends).

Most classes captured similar patterns of physical activity and sedentary behaviour on weekdays and weekends, apart from the *Highly active* classes which contained children who are more active and less sedentary at the weekend than during the week. The two *Active* classes were similar in terms of MVPA but differed in time spent in sedentary and light activity, and likewise for the two *Inactive* classes. In the *Highly active* and *Active* classes, the majority met the recommendation of 60+ mins of MVPA, with more achieving the guidelines at weekends than on weekdays. Only a minority of those in the *Inactive* classes achieved the recommended MVPA levels, with this proportion higher on weekdays than weekends.

#### Associations with class membership

The *Highly active* classes were predominantly boys (88%), while the *Inactive/sed* class comprised 60% girls (Table 1). There were similar proportions of girls and boys in the other classes. The *Inactive/light* class had a higher level of deprivation (IMD score = 18.9 95% CI: 15.7, 22.2) than other classes. BMI z-score at ages 6 or 9 and average hours of screen viewing were similar across the classes (Table 1).

**Table 1:** Age 6: Model-based estimates of covariate means and test for differences across classes

	Female	BMI z-score (age 6)		IMD score (age 6)		Total screen viewing (hrs)	
	%	Mean	95% CI	Mean	95% CI	Mean	95% CI
OVERALL	47.6%	0.23		14.6		2.36	
Highly active	12.1%	0.27	(−0.04, 0.57)	12.8	(9.47, 16.13)	2.14	(1.85, 2.42)
Active/ light	48.9%	0.45	(0.22, 0.67)	14.5	(12.38, 16.67)	2.37	(2.14, 2.59)
Active/ sed	46.2%	−0.12	(−0.41, 0.18)	13.2	(10.88, 15.55)	2.67	(2.41, 2.94)
Inactive/light	53.3%	0.36	(0.02, 0.70)	18.9	(15.66, 22.22)	2.25	(2.00, 2.49)
Inactive/sed	60.1%	0.33	(0.11, 0.54)	13.8	(11.77, 15.79)	2.30	(2.09, 2.51)
*P*-value^a^	< 0.0005	0.052		0.034		0.110	

^a^Wald test for differences in means across latent classes

### Age 9 cross-sectional latent profiles

The BIC for the data (Additional file 1: Table S2) indicated either a 6 or 7-class model, with the LMR test giving support to a 6-class model. The 7-class model has a slightly lower BIC but produced one very small class (1%) of highly sedentary and inactive children, similar to that observed for the age 6 data, and we encountered problems with model identifiability due to the small numbers. As the remaining six classes had very similar profiles to the 6-class model, we chose this model, consistent with our approach for the age 6 data.

**Fig. 2:**
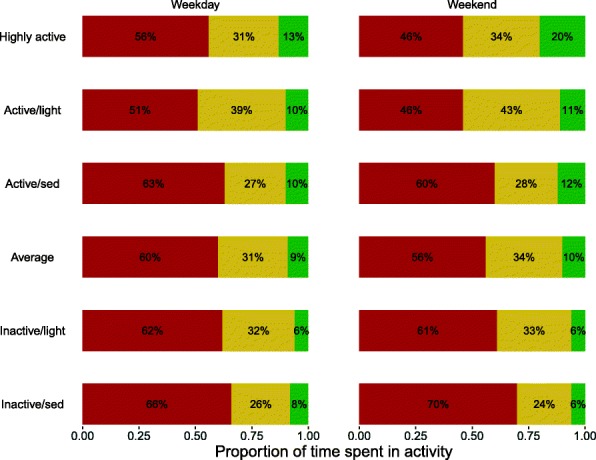
Class profiles for Age 9. Proportions of time spent in sedentary (red), light (yellow) and MVPA (green) at weekdays and weekends for the 6 classes identified at age 9. Classes are ordered roughly from most active to least active. While class labels are similar to those used for age 6, there are some differences – see text for details

When labelling the Age 9 classes, we used some of the Age 6 labels to reflect similar profiles. However, these classes are not directly equivalent as nearly all classes are more sedentary at age 9 than at age 6. Instead we have defined ‘similar’ in terms of patterns of high and low activity and sedentary proportions, when compared to the overall year average levels. This means, for example, that the *Inactive/sed* class is always defined as being less active and more sedentary than average, however the actual proportion of time spent in sedentary behaviour in this class is higher at age 9 than at age 6 while MVPA remains similar. We have highlighted differences in interpretation of classes below, and the reader is encouraged to keep these differences in mind when making comparisons. The class profiles are summarised in Additional file 1: Table S3 and Fig. 2, and were given the following labels:

- **Highly active** (7%): Very similar to the Age 6 *Highly active* class. High and very high levels of MVPA, especially at weekends, combined with low sedentary time. Nearly all meet the recommended MVPA (90% on weekdays and 100% on weekends)
- **Active/light** (6%): Similar to the Age 6 *Active/light* class, but with slightly lower sedentary proportions. Higher MVPA than average and average to low sedentary – more so at weekends. A large proportion of non-sedentary time is spent in light activity rather than MVPA. Most meet the recommended MVPA (68% on weekdays and 78% on weekends)
- **Active/sed** (11%): Very similar to the *Active/sed* class at age 6. Higher MVPA than average (similar to *Active/light*), but above average sedentary times – more so at weekends. Most meet the recommended MVPA (72% on weekdays and 91% on weekends)
- **Average** (33%): No corresponding class at age 6. Average levels of sedentary and MVPA; slightly more active and less sedentary at weekends. The majority met the recommended MVPA (59% on weekdays and 73% on weekends).
- **Inactive/light** (22%): Similar pattern to the Age 6 *Inactive/light* class, but MVPA is much lower. Very low MVPA combined with average sedentary; a large proportion of non-sedentary time is spent in light activity. A minority meet the recommended MVPA (11% on weekdays and 11% on weekends)
- **Inactive/sed** (21%): Similar to the Age 6 *Inactive/sed* class. Low/ very low MVPA (similar to *Inactive/light)* but combined with very high sedentary proportions. A minority meet the recommended MVPA (42% on weekdays and 14% on weekends)

In contrast to the classes at age 6, we saw slightly stronger differences in weekday/weekend patterns of physical activity and sedentary time within the classes, with all classes except *Inactive/sed* exhibiting lower sedentary proportions at weekends. The majority of children in the *Highly Active*, *Active/light*, *Active/sed* and *Average* classes met the recommended levels of MVPA, especially at weekends. Only a minority of those in the two *Inactive* classes met the recommended MVPA levels, with this proportion higher on weekdays than weekends.

#### Associations with class membership

At age 9, all covariates differ between classes. Most participants in the *Highly active* class were boys (93%) whereas most in the *Inactive/light* class were girls (75%; Table 2). Differences in BMI z-score are driven by the high value for the *Active/light* class and low value for *Active/sed.* The *Active/light* class has a higher IMD score, and so contains more individuals with higher deprivation scores than other classes. Average hours of screen viewing differed between classes, and this was mainly due to differences in weekend screen viewing (Additional file 1: Table S4); higher levels of weekend screen viewing were seen in the two *Inactive* classes, and also in the *Highly active* class. These average approximately 4 h of screen-viewing at weekends, compared to 3–3.5 h for the other classes. There was a strong association between class membership and activity participation, with a difference between the lowest activity in the *Inactive/sed* class and the highest in the *Highly active* class of 2.4, which corresponds approximately to an extra four to five sessions of activity per week. When the type of activity was further separated into participation in structured (clubs) and unstructured (playing outdoors) activities (Additional file 1: Table S4) we saw a different pattern in the two *Active* classes, with *Active/sed* higher in structured activity and *Active/light* higher in unstructured. Those in the *Highly active* class score high on both.

**Table 2:** Age 9: Model-based estimates of covariate means and test for differences across classes

	% female	BMI z-score (age 9)		IMD score (age 9)		Total screen viewing (hrs)		Activity participation	
	%	Mean	95% CI	Mean	95% CI	Mean	95% CI	Mean	95% CI
OVERALL	55.2%	0.32		15.6		2.89		5.87	
Highly active	7.1%	0.22	(−0.02, 0.46)	15.3	(11.5, 19.1)	2.90	(2.46, 3.34)	7.53	(6.91, 8.14)
Active/ light	44.2%	0.81	(0.42, 1.19)	21.8	(15.5, 28.1)	2.33	(1.78, 2.88)	6.25	(5.45, 7.04)
Active/ sed	44.6%	−0.18	(−0.43, 0.07)	11.8	(9.1, 14.5)	2.72	(2.35, 3.10)	6.14	(5.56, 6.72)
Average	56.2%	0.27	(0.13, 0.42)	15.5	(13.4, 17.5)	2.70	(2.46, 2.93)	6.11	(5.78, 6.44)
Inactive/light	75.1%	0.56	(0.36, 0.75)	17.8	(15.2, 20.5)	3.03	(2.71, 3.35)	5.38	(5.00, 5.77)
Inactive/sed	55.2%	0.36	(0.16, 0.56)	14.6	(12.2, 17.0)	3.28	(3.00, 3.55)	5.13	(4.74, 5.52)
*P*-value^a^	< 0.0005	< 0.0005		0.007		0.007		< 0.0005	

^a^Wald test for differences in means across latent classes

### Changes between profiles at age 6 and age 9

#### Comparison of age 6 and age 9 profiles

**Fig. 3:**
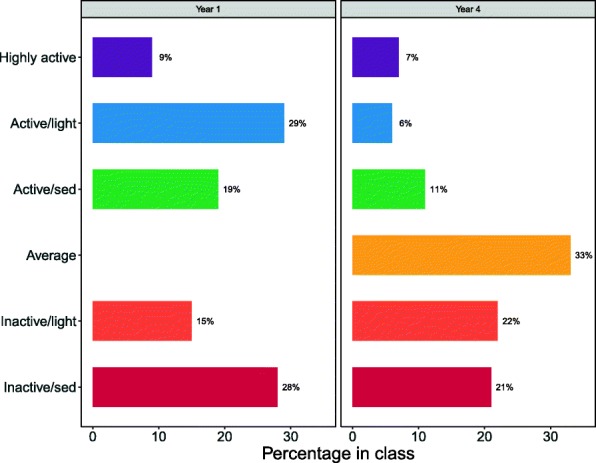
Comparison of class prevalence between ages 6 (left) and 9 (right). Classes with similar labels show similar patterns at ages 6 and 9, but there are some differences; see text for details. Remaining classes are seen only in the given year

Figure 3 shows the prevalence of the different classes at age 6 and age 9, side by side. Fewer children at age 9 are members of the *Active* classes than at age 6, especially the *Active/light*, and the new class, *Average*, is the most commonly occurring class. The proportion of children in the two *Inactive* classes is approximately the same, but the *Inactive/sed* class has reduced and *Inactive/light* increased. MVPA was much lower in the Age 9 *Inactive/light* class than either of the Age 6 *Inactive* classes.

#### Transition between ages 6 and 9

We investigated fixing the same latent classes at both time points (measurement invariance model), but this did not fit the data well. This is supported by the cross-sectional findings, which found a different number of latent classes at each age and differences in the class definitions, in particular with higher sedentary proportions at age 9. The results presented here are based on a non-invariance model (classes differ), with latent class profiles fixed to be the same as those found in the cross-sectional analyses.

**Fig. 4:**
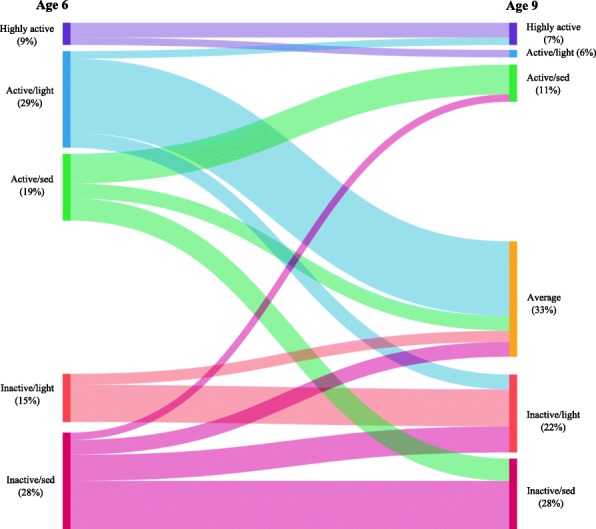
Transition between classes at age 6 and 9. All classes at age 9 are more sedentary than their similarly-named counterparts at age 6; see text for details. Small transitions (< 2%) have been omitted for clarity

Figure 4 shows how children moved between Age 6 classes and Age 9 classes using the model-based estimates of the transition probabilities (Additional file 1: Table S5). There was substantial movement between classes between ages 6 and 9, with around 30% remaining in the similar class. Note that with the exception of the *Active/light* class, all Age 9 classes were more sedentary than their Age 6 counterparts, and so children who move to a more active class may still become more sedentary. The most common patterns of movement were to classes with either no change or a decrease in MVPA (84%), and children in active classes at age 6 were more likely to remain in active classes at age 9 (45%) than children in inactive classes at age 6 were to move to active classes at age 9 (12%). Children who move from *Active* to *Inactive* classes tended to keep the same light/sedentary behaviour, while children who moved away from *Highly active* tended to move to the light rather sedentary classes.

The transition probabilities between classes were associated with all the characteristics that we compared, except deprivation score. Girls had higher probability of moving to classes with lower MVPA than boys (Additional file 1: Table S5). Additional file 1: Figures S1-S3 show how the transition probabilities depend on BMI z-score at age 6, activity participation score and hours of weekend screen viewing. Higher BMI z-score at age 6 was associated with lower probabilities of moving to more active classes and higher probabilities of moving to less active classes. For example, overall, children in the *Active/sed* class at age 6 had a 43% probability of staying in the similar Age 9 class and 30% probability of moving to *Inactive/sed*. For children with a BMI z-score of 1 (corresponding roughly to a definition of ‘Overweight’ [31]) these transitions are estimated at 29% and 38%.

The probability of remaining in the *Highly active* class between ages 6 and 9 is strongly associated with higher activity participation. A child in the *Highly active* class at age 6 with a participation score at the average of 6 has a 32% probability of remaining in the *Highly active* class, increasing by approximately 8 percentage points for every extra activity session per week (one unit increase in participation score). A higher participation score is also associated with an increase in the probability of moving into the *Highly active* class at age 9 from other classes. Activity participation was also associated with moving from *Inactive* classes to *Active* classes, although the numbers of children transitioning between these two was small. Patterns were similar for associations with structured and unstructured activity (not shown), but with high levels of unstructured activity and low levels of structured activity associated with a move to the *Active/light* class, and high levels of structured activity associated with movement to the *Active/sed* class.

Transition probabilities were associated with the average number of hours spent screen viewing at weekends but not on weekdays. The strongest patterns were for the *Active/sed* and *Inactive/sed* classes at age 6 where more hours spent screen viewing increased the probability of transitioning to the *Inactive/sed* class at age 9. These probabilities are 31% for *Active/sed* and 47% for *Inactive/sed* for an average screen viewing of 3.8 h at weekends, increasing to 41% and 58% for an extra 2 h. In the *Highly active* class, the opposite pattern was seen, with very high screen-viewing associated with remaining in the *Highly active* class.

## Discussion

The data presented in this paper have shown that five different classes described the physical activity and sedentary behaviour of pupils at age 6 and six classes at age 9. The profiles range from very active to inactive with differentiation between subgroups in terms of sedentary and light behaviour. Five classes were found to be similar between the 2 years, in terms of general patterns, with the addition of a substantial extra class of ‘average’ children at age 9 who fell between the more extreme active and inactive classes seen at age 6. This suggests that the classes are relatively stable, although the proportions in each class change, with the active classes decreasing in prevalence. It is important to note that consistent with a previous study using similar methods applied to physical activity data from several countries [4], all classes were more sedentary at age 9 than at age 6 and that the classes picked up important differences between weekday and weekend physical activity which would not have been apparent by comparing average time in MVPA. Of relevance is that the active classes tended to engage in the recommended 60 min of MVPA at the weekend, more so than during the week, while inactive classes were unlikely to meet this guideline at either weekends or week days. Recent research has shown that adults who engage in high levels of physical activity at the weekend but are less active on weekdays (i.e., weekend warriors) have reduced risk of cardiovascular disease and all-cause mortality [46], suggesting that weekend activity may be important for health outcomes in adults. Some studies have assessed weekday and weekend differences in children [47], but none have looked at the contribution weekend activity makes towards overall MVPA recommendations. Our results suggest that the contribution of weekend activity to an average of 60 min per day of MVPA across the week [48] among children may warrant further investigation.

All Age 9 classes were more sedentary than their Age 6 counterparts. There was substantial movement between classes between age 6 and 9 with less than a third of pupils remaining in the similar class across the two time points. Across this time, the strongest patterns of movement were to profiles with either no change or a decrease in MVPA, and even where children moved to a more active class they typically become more sedentary. This highlights a need to understand the factors that are associated with change in activity profile from age 6 to 9 and how to mitigate movement to less active profiles.

The most robust finding from previous studies is an association between girls and low-activity clusters [22] and this is supported by our findings. Specifically, the *Highly active* class is predominantly boys at both ages, with the proportion of boys increasing from 88 to 93%. Likewise, at the other end of the scale the *Inactive* classes have a higher proportion of girls. Gender is also strongly associated with movement between classes with girls more likely to move to less active classes and boys more likely to move to more active classes. Unfortunately, sample sizes were too small to produce separate profiles for boys and girls, especially to look at change over time or associations with covariates. However, the findings do suggest that girls may particularly be at risk of moving to less active profiles.

Participants’ body mass-index (z-score), screen-viewing behaviour and the extent to which they engaged in activities were associated with movement between classes. These characteristics were not strongly associated with class membership at age 6 but showed much stronger links with classes at age 9. Movement to more active classes was associated with a lower BMI z-score at age 6 and higher activity participation, with the latter strongly associated with movement into the *Highly active* class. This indicates that both structured and unstructured activities may contribute to children’s overall physical activity levels and maintained engagement in such activities may mitigate some of the age-related decline in activity. Conversely, screen-viewing was associated with becoming more sedentary, rather than becoming less active. For example, in the *Highly active* class, more screen-viewing was associated with remaining *Highly active*, suggesting that for this class, screen-viewing occurs in addition to, rather than instead of, being active. This is consistent with a male ‘techno-active’ group commonly identified in cluster analyses [49]. Collectively, these changes in class membership highlight how screen-viewing, body mass and the types of activities in which children engage can affect their overall physical activity profile. There is strong evidence that sedentary behaviour tracks from childhood to adulthood [50] and that sedentary behaviour is associated with adverse health impacts in adults [7]. The findings therefore suggest that gaining a detailed understanding of the nature of these associations may be important for developing more effective behaviour change programs.

A key finding from this study was that children who are active are more likely to remain active than inactive children to become active. For example, children in the *Active/light* and *Active/sed* classes at age 6 have similar MVPA but have distinct patterns of movement. Most of the *Active/light* class move to the new *Average* class at age 9; their proportion of non-sedentary time spend in MVPA remains the same but as sedentary time increases, the total MVPA decreases. Meanwhile, children in the *Active/sed* class are most likely to either remain in *Active/sed* (more sedentary time, but similar MVPA) or move to *Inactive/sed* (more sedentary time and less MPVA). In both cases, those who move to *Inactive* classes tend to keep the same light/sedentary behaviour. This suggests that sedentary patterns reflect underlying behaviour preferences which are independent of how much activity children engage in. This finding suggests that early physical activity behaviours may contribute to physical activity throughout childhood and developing strategies to engage children in physical activity early and then keep them active are likely to be very important. Research that examines this possibility is therefore essential for the advancement of the field.

The differences in movement from the *Active/light* and *Active/sed* classes may be due to changes in the amount of structured versus unstructured activity, with parents reporting a decrease in free play between age 6 and 9 [51]. While an activity participation score is not available at age 6, if we assume the classes have similar activity profiles as at age 9, a key difference between *Active/light* and *Active/sed* is that the former class engage in predominantly unstructured activity while the latter in structured activity. A gradual decline in the amount of unstructured activity, when not replaced by structured activities results in a decline in activity levels (i.e., *Active/light* to the *Average* class). Conversely, those in the *Active/sed* class may either continue their club activity and remain in *Active/sed*, or experience a sudden drop in MVPA when they stop the activity and they transition to the *Inactive/sed* class. In addition, we note that both *Active/sed* and *Inactive/sed* are more likely at age 9 to meet the recommended 60 min of MVPA than their light counterparts, despite having similar MVPA and engaging in more sedentary time. This suggests that unstructured activities may be more variable in the intensity of activity (perhaps more often dipping into light rather than moderate activity), whereas structured activity may enable children to more consistently meet MVPA guidelines. This highlights the potential important role of structured activities, such as after-school activity clubs, in promoting and maintaining physical activity in childhood, and if unstructured activity continues to decline may indicate that those in the *Average* and *Active/light* classes are at risk of becoming more inactive in the future.

### Strengths and limitations

The major strength of this study is the use of objective physical activity and sedentary behaviour data to examine how physical activity profiles change from Year 1 (age 6) to Year 4 (age 9) of primary school. By applying latent profile analysis to a contextually rich dataset we have provided new information which provides some insights into the change in profiles and the potentially important role of weekend physical activity. The study does however have several limitations that need to be considered. Although the data are from a relatively large cohort, the sample is from a single UK city and some of the transitions are based on a relatively small number of cases. As a result, we are unable to conduct separate analyses for boys and girls, despite the strong associations between gender and latent classes. The class grouping was relatively consistent between the two time points, which suggests a degree of robustness, but the latent class approach is data driven which makes comparisons between studies more difficult. Finally, it is also important to recognise that the move from Year 1 to Year 4 is a key period of change in children’s lives when their motor skills develop, they get increased licence to be physically active and there are numerous other social changes, such as going to bed later and increased homework, which can all impact on physical activity and sedentary time [51]. These broader factors may be associated with the profiles detected at the two time points and, aside from the reasons discussed, may help to explain movement in classes across the two timepoints.

## Conclusions

Five profiles were identified at ages 6 and six profiles at age 9, reflecting different patterns of physical activity and sedentary time, and differences between weekdays and weekends. There was substantial movement between profiles between ages 6 and 9, with transitions associated with sex, BMI z-score, screen-viewing and participation in out-of-school activities. Our results highlight the importance of engaging children in physical activity early and the potential important role of structured activities, such as after-school activity clubs, in promoting and maintaining physical activity in childhood. Weekend differences suggest that greater focus on how weekend activity contributes to an average of 60 min per day of MVPA across the week may be warranted.

## Additional file

Additional file 1: **Table S1.** Characteristics of sample – observed and missing data. **Table S2**. Model Fit for models with 2–10 classes. **Table S3.** Class membership proportions and percentage time in sedentary, light and MVPA respectively. **Table.** Age 9: Model-based estimates of additional covariate means and test for differences across classes. **Table S5.** Estimated transition probabilities: probability a child will move from a profile at age 6 to a profile at age 9. **Figure S1**. Associations between transition probabilities and BMI z-score at age 6. How transition probabilities from classes at age 6 (panels) to classes at age 9 (lines) change with BMI z-score at age 6. **Figure S2.** Associations between transition probabilities and activity participation. How transition probabilities from classes at age 6 (panels) to classes at age 9 (lines) change with activity participation score. A one unit increase in activity score corresponds to approximately one extra session of activity per week. **Figure S3.** Associations between transition probabilities and weekend screen-viewing. How transition probabilities from classes at age 6 (panels) to classes at age 9 (lines) change with hours of weekend screen-viewing. (DOCX 397 kb)

## Abbreviations

BIC: Bayesian Information Criterion
BLRT: Bootstrapped likelihood ratio test
BMI: Body-mass index
IMD: Indices of Multiple Deprivation
LCA: Latent class analysis
LMR: Lo-Mendell-Rubin test
LPA: Latent profile analysis
MVPA: Moderate-to-vigorous-intensity physical activity
