# Supplementary material for: Profiles of children’s physical activity and sedentary behaviour between age 6 and 9: a latent profile and transition analysis
Source: Int J Behav Nutr Phys Act. 2018 Oct 23;15:103. doi: 10.1186/s12966-018-0735-8 (PMC6199754; doi:10.1186/s12966-018-0735-8)

**Additional file 1**

**Additional Tables**

**Table S1:** Characteristics of sample – observed and missing data.

**Table S2:** Model Fit for models with 2-10 classes.

**Table S3:** Class membership proportions and percentage time in sedentary, light and MVPA respectively.

**Table S4:** Age 9: Model-based estimates of additional covariate means and test for differences across classes.

**Table S5:** Estimated transition probabilities: probability a child will move from a profile at age 6 to a profile at age 9.

**Additional Figures**

**Figure S1: Associations between transition probabilities and BMI z-score at age 6.** How transition probabilities from classes at age 6 (panels) to classes at age 9 (lines) change with BMI z-score at age 6.

**Figure S2:** **Associations between transition probabilities and activity participation**. How transition probabilities from classes at age 6 (panels) to classes at age 9 (lines) change with activity participation score. A one unit increase in activity score corresponds to approximately one extra session of activity per week.

**Figure S3:** **Associations between transition probabilities and weekend screen-viewing.** How transition probabilities from classes at age 6 (panels) to classes at age 9 (lines) change with hours of weekend screen-viewing.

**Table S1: Characteristics of sample – observed and missing data**

|  |  | YEAR 1 (Age 6)  N=1201 | | |  | YEAR 4 (Age 9)  N=1167 | | |
| --- | --- | --- | --- | --- | --- | --- | --- | --- |
|  |  | mean | sd | Missing  n (%) |  | mean | Sd | Missing  n (%) |
| Weekday | |  |  |  |  |  |  |  |
|  | MVPA (mins) | 68.0 | 21.1 | 114 (9%) |  | 62.3 | 22.4 | 90 (8%) |
|  | Light activity (mins) | 244.2 | 39.9 | 114 (9%) |  | 222.1 | 43.6 | 90 (8%) |
|  | Sedentary (mins) | 372.0 | 59.3 | 114 (9%) |  | 445.7 | 64.5 | 108 (9%) |
|  | Proportion of MVPA | 0.10 | 0.03 | 114 (9%) |  | 0.08 | 0.03 | 90 (8%) |
|  | Proportion of light activity | 0.36 | 0.05 | 114 (9%) |  | 0.30 | 0.06 | 90 (8%) |
|  | Proportion of sedentary time | 0.54 | 0.06 | 114 (9%) |  | 0.61 | 0.06 | 108 (9%) |
| Weekend | |  |  |  |  |  |  |  |
|  | MVPA (mins) | 66.3 | 27.6 | 221 (18%) |  | 61.3 | 32.0 | 207 (18%) |
|  | Light activity (mins) | 234.0 | 46.6 | 221 (18%) |  | 214.8 | 51.0 | 207 (18%) |
|  | Sedentary (mins) | 339.1 | 76.0 | 221 (18%) |  | 401.9 | 83.7 | 225 (10%) |
|  | Proportion of MVPA | 0.10 | 0.04 | 221 (18%) |  | 0.09 | 0.05 | 207 (18%) |
|  | Proportion of light activity | 0.37 | 0.06 | 221 (18%) |  | 0.31 | 0.07 | 207 (18%) |
|  | Proportion of sedentary time | 0.53 | 0.08 | 221 (18%) |  | 0.59 | 0.09 | 225 (10%) |
| % meeting 60 mins avg MVPA /day | | 62.7% |  | 0 (0%) |  | 46.9% |  | 0 (0%) |
| Sex (% female) | | 47.6% |  | 0 (0%) |  | 55.2% |  | 0 (0%) |
| Standardised BMI z-score | | 0.23 | 0.92 | 206 (17%) |  | 0.32 | 1.06 | 3 (<1%) |
| IMD score | | 14.6 | 12.6 | 83 (7%) |  | 15.6 | 13.9 | 14 (1%) |
| Weekday screen viewing (hrs) | | 1.69 | 1.13 | 166 (14%) |  | 1.96 | 1.29 | 210 (18%) |
| Weekend screen viewing (hrs) | | 3.05 | 1.64 | 170 (14%) |  | 3.83 | 2.04 | 211 (18%) |
| Total screen viewing (hrs) | | 2.36 | 1.22 | 177 (15%) |  | 2.89 | 1.50 | 212 (18%) |
| Participation in clubs^1^ | |  |  |  |  | 2.25 | 1.38 | 9 (1%) |
| Participation in free play^1^ | |  |  |  |  | 3.60 | 1.61 | 29 (2%) |
| Activity participation score^1^ | |  |  |  |  | 5.87 | 2.27 | 29 (2%) |

^1^Not recorded in Year 1

**Table S2: Model Fit for models with 2-10 classes**

| Classes | BIC^1^ | Log-likelihood | LMR^2^ | BLRT^2^ | Smallest class size | Relative  Entropy |
| --- | --- | --- | --- | --- | --- | --- |
| **Year 1 (Age 6)** | | | | | | |
| 2 | -14497.2 | 7297.803 | <0.0005 | <0.0005 | 33% | 0.612 |
| 3 | -14621.1 | 7384.403 | 0.0022 | <0.0005 | 25% | 0.559 |
| 4 | -14663.7 | 7430.295 | 0.1729 | <0.0005 | 18% | 0.562 |
| 5 | -14683.7 | 7464.894 | 0.2740 | <0.0005 | 9% | 0.574 |
| 6 | -14673.6 | 7484.456 | 0.5110 | <0.0005 | 6% | 0.581 |
| 7 | -14656.6 | 7500.584 | 0.2061 | 0.0300 | 6% | 0.580 |
| 8 | -14633.6 | 7513.687 | 0.0304 | 0.0128 | 2% | 0.610 |
| 9 | -14607.3 | 7525.154 | 0.3341 | 1.0000 | 2% | 0.635 |
| 10 | -14580.9 | 7536.561 | 0.0363 | 0.0923 | 1% | 0.637 |
| **YEAR 4 (Age 9)** | | | | | | |
| 2 | -13857.4 | 6977.831 | 0.0001 | <0.0005 | 25% | 0.637 |
| 3 | -14037.6 | 7092.503 | <0.0005 | <0.0005 | 12% | 0.640 |
| 4 | -14140.0 | 7168.318 | 0.0039 | <0.0005 | 11% | 0.618 |
| 5 | -14167.4 | 7206.560 | 0.4917 | <0.0005 | 11% | 0.618 |
| 6 | -14171.5 | 7229.255 | <0.0005 | <0.0005 | 9% | 0.619 |
| 7 | -14186.8 | 7255.034 | 0.1396 | <0.0005 | 5% | 0.632 |
| 8 | -14167.7 | 7280.457 | 0.2397 | <0.0005 | 31% | 0.663 |
| 9 | -14148.6 | 7295.487 | 0.2396 | <0.0005 | 1% | 0.643 |
| 10 | -14137.2 | 7314.553 | 0.1077 | <0.0005 | 1% | 0.649 |

^1^ lower BIC indicates better model fit

^2^ p-value for test comparing the current number of classes to a model with one fewer classes

**Table S3: Class membership proportions and percentage time in sedentary, light and MVPA respectively**

|  | Age 6 (n=1132)  (including high sed outliers) | | | Age 6 (n=1128) | | | | | | Age 9 (n=1121) | | | | |
| --- | --- | --- | --- | --- | --- | --- | --- | --- | --- | --- | --- | --- | --- | --- |
|  |  |  |  |  |  |  | >60mins^3^ | | |  |  |  | >60mins^3^ | |
| Class | Prop^1^ | Wday^2^ | Wkend^2^ | Prop^1^ | Wday^2^ | Wkend^2^ | wday | | wkend | Prop^1^ | Wday^2^ | Wkend^2^ | wday | wkend |
| Highly active | 9% | 50%  37%  13% | 42%  39%  19% | 9% | 50%  37%  13% | 43%  39%  18% | 92% | 100% | | 7% | 56%  31%  13% | 46%  34%  20% | 90% | 100% |
| Active/ light | 28% | 51%  38%  11% | 48%  40%  12% | 29% | 52%  37%  11% | 48%  40%  12% | 77% | 87% | | 6% | 51%  39%  10% | 46%  43%  11% | 68% | 78% |
| Active/ sed | 18% | 56%  33%  11% | 56%  33%  11% | 19% | 56%  33%  11% | 57%  31%  12% | 77% | 86% | | 11% | 63%  27%  10% | 60%  28%  12% | 72% | 91% |
| Average |  |  |  |  |  |  |  |  | | 33% | 60%  31%  9% | 56%  34%  10% | 59% | 73% |
| Inactive/light | 15% | 54%  38%  8% | 51%  41%  8% | 15% | 54%  38%  8% | 51%  41%  8% | 33% | 19% | | 22% | 62%  32%  6% | 61%  33%  6% | 11% | 11% |
| Inactive/ sed | 28% | 58%  33%  9% | 59%  34%  7% | 28% | 58%  34%  8% | 60%  33%  7% | 33% | 20% | | 21% | 66%  26%  8% | 70%  24%  6% | 42% | 14% |
|  | 1% | 62%  32%  6% | 68%  27%  5% |  |  |  |  |  | |  |  |  |  |  |

^1^ Estimated proportion in each class

^2^ From top to bottom: % of total time spent in sedentary, light and MVPA

^3^ Estimated proportion in class who meet the recommended MVPA of 60 minutes or more per day

**Table S4: Age 9: Model-based estimates of additional covariate means and test for differences across classes**

|  | Weekday screen viewing (hrs) | | Weekend screen viewing (hrs) | | Structured activity participation | | Unstructured activity participation | |
| --- | --- | --- | --- | --- | --- | --- | --- | --- |
|  | 95% CI | | 95% CI | | 95% CI | | 95% CI | |
| OVERALL | 1.96 |  | 3.83 |  | 2.25 |  | 3.60 |  |
| Highly active | 1.96 | (1.57, 2.34) | 3.84 | (3.26, 4.42) | 3.04 | (2.61, 3.47) | 4.44 | (4.05, 4.83) |
| Active/ light | 1.64 | (1.13, 2.16) | 3.02 | (2.33, 3.70) | 1.87 | (1.33, 2.41) | 4.33 | (3.75, 4.91) |
| Active/ sed | 1.82 | (1.48, 2.16) | 3.63 | (3.13, 4.12) | 2.63 | (2.27, 2.98) | 3.52 | (3.12, 3.92) |
| Average | 1.84 | (1.64, 2.03) | 3.58 | (3.25, 3.90) | 2.37 | (2.17, 2.56) | 3.75 | (3.51, 3.99) |
| Inactive/light | 2.13 | (1.85, 2.40) | 3.94 | (3.52, 4.36) | 2.01 | (1.78, 2.24) | 3.35 | (3.07, 3.63) |
| Inactive/sed | 2.16 | (1.91, 2.40) | 4.40 | (4.01, 4.79) | 1.95 | (1.71, 2.19) | 3.18 | (2.90, 3.45) |
| P-value^1^ | 0.17 |  | 0.005 |  | <0.0005 |  | <0.0005 |  |

^1^Wald test for differences in means across latent classes.

**Table S5: Estimated transition probabilities: probability a child will move from a profile at age 6 to a profile at age 9**

|  | Age 9 Profile | | | | | |  |
| --- | --- | --- | --- | --- | --- | --- | --- |
|  | Highly active | Active/ light | Active/ sed | Average | Inactive/light | Inactive/sed |  |
| Age 6 Profile |  |  |  |  |  |  |  |
| Highly active | 0.39 | 0.21 | 0.04 | 0.16 | 0.15 | 0.05 |  |
| Active/ light | 0.08 | 0.05 | 0.02 | 0.70 | 0.12 | 0.03 |  |
| Active/ sed | 0.07 | 0.01 | 0.43 | 0.19 | 0.00 | 0.30 |  |
| Inactive/light | 0.02 | 0.05 | 0.03 | 0.21 | 0.67 | 0.02 |  |
| Inactive/sed | 0.00 | 0.05 | 0.08 | 0.14 | 0.25 | 0.48 |  |
| **Boys** | | | | | | |  |
| Highly active | 0.59 | 0.20 | 0.05 | 0.11 | 0.06 | 0.08 |  |
| Active/ light | 0.16 | 0.06 | 0.07 | 0.63 | 0.07 | 0.02 |  |
| Active/ sed | 0.11 | 0.02 | 0.48 | 0.13 | 0.00 | 0.26 |  |
| Inactive/light | 0.05 | 0.08 | 0.01 | 0.33 | 0.51 | 0.02 |  |
| Inactive/sed | 0.00 | 0.05 | 0.12 | 0.14 | 0.09 | 0.61 |  |
| **Girls** | | | | | | |  |
| Highly active | 0.20 | 0.25 | 0.03 | 0.21 | 0.31 | 0.09 |  |
| Active/ light | 0.03 | 0.05 | 0.03 | 0.69 | 0.20 | 0.01 |  |
| Active/ sed | 0.03 | 0.03 | 0.35 | 0.28 | 0.00 | 0.31 |  |
| Inactive/light | 0.00 | 0.03 | 0.00 | 0.18 | 0.77 | 0.01 |  |
| Inactive/sed | 0.00 | 0.04 | 0.05 | 0.17 | 0.30 | 0.43 |  |
|  |  |  |  |  |  |  |  |

**Figure S1: Associations between transition probabilities and BMI z-score at age 6.** How transition probabilities from classes at age 6 (panels) to classes at age 9 (lines) change with BMI z-score at age 6.


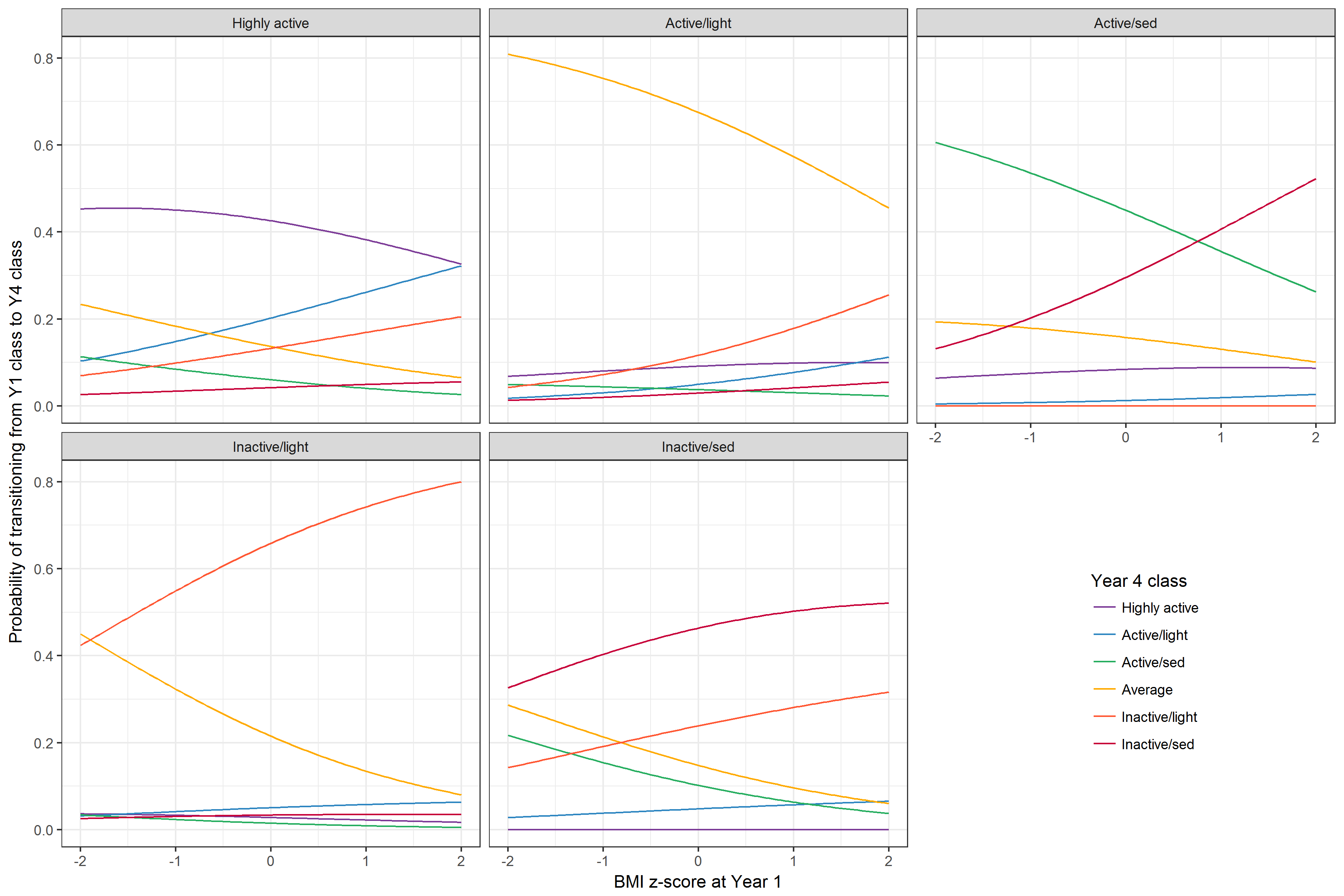


**Figure S2:** **Associations between transition probabilities and activity participation**. How transition probabilities from classes at age 6 (panels) to classes at age 9 (lines) change with activity participation score. A one unit increase in activity score corresponds to approximately one extra session of activity per week.


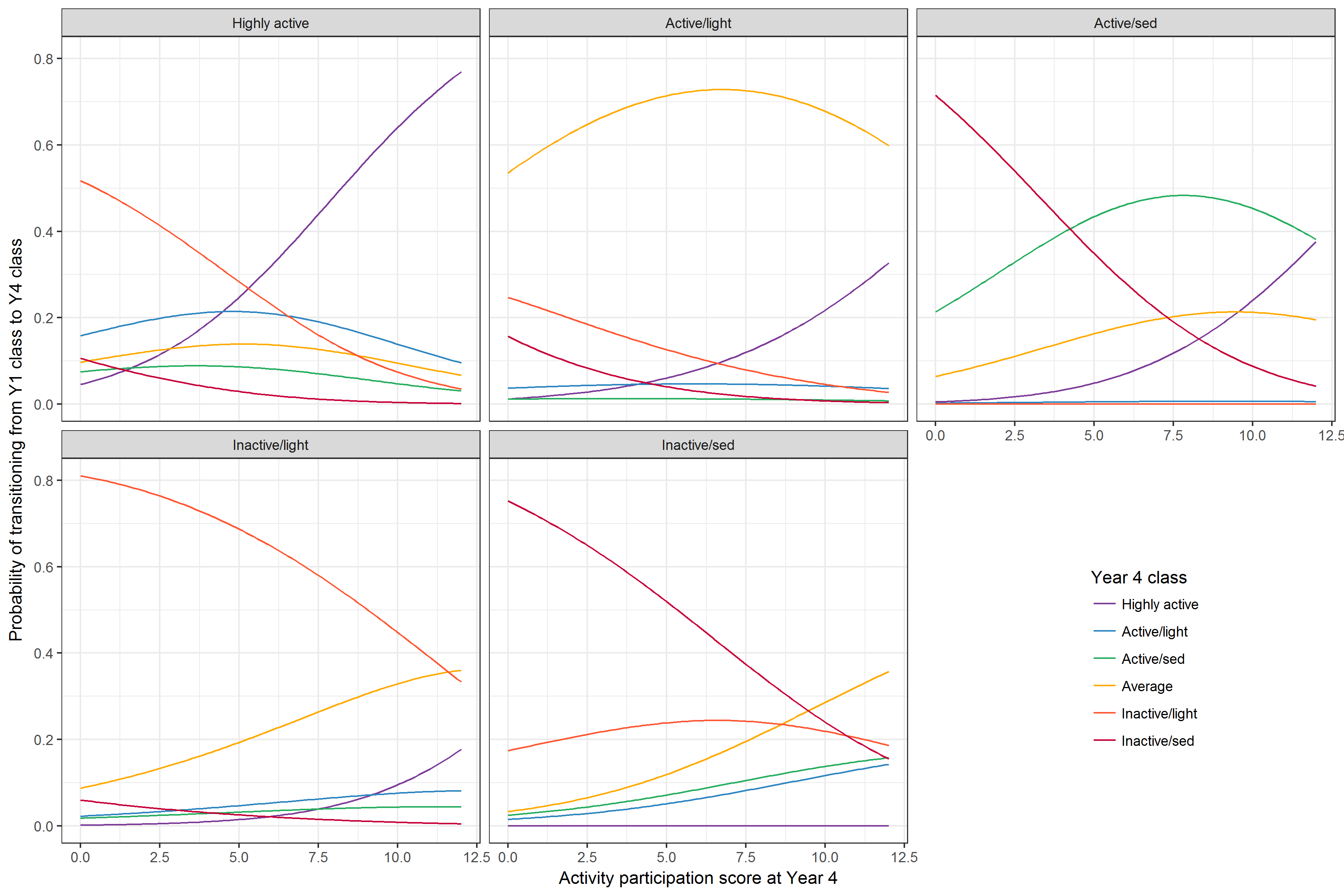


**Figure S3:** **Associations between transition probabilities and weekend screen-viewing.** How transition probabilities from classes at age 6 (panels) to classes at age 9 (lines) change with hours of weekend screen-viewing.


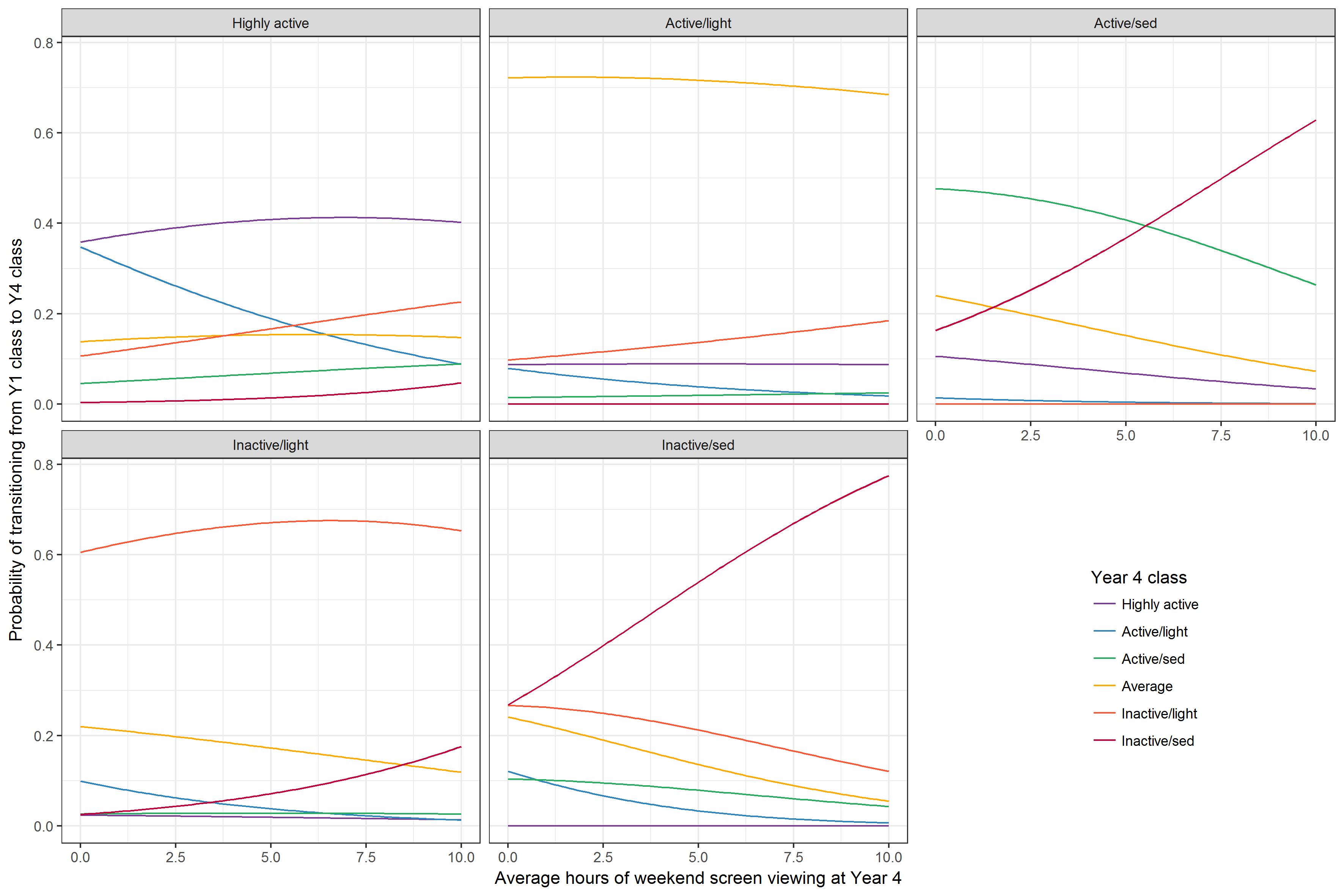

Supplement: Supplementary file 1 — Table S1. Characteristics of sample – observed and missing data. Table S2. Model Fit for models with 2–10 classes. Table S3. Class membership proportions and percentage time in sedentary, light and MVPA respectively. Table. Age 9: Model-based estimates of additional covariate means and test for differences across classes. Table S5. Estimated transition probabilities: probability a child will move from a profile at age 6 to a profile at age 9. Figure S1. Associations between transition probabilities and BMI z-score at age 6. How transition probabilities from classes at age 6 (panels) to classes at age 9 (lines) change with BMI z-score at age 6. Figure S2. Associations between transition probabilities and activity participation. How transition probabilities from classes at age 6 (panels) to classes at age 9 (lines) change with activity participation score. A one unit increase in activity score corresponds to approximately one extra session of activity per week. Figure S3. Associations between transition probabilities and weekend screen-viewing. How transition probabilities from classes at age 6 (panels) to classes at age 9 (lines) change with hours of weekend screen-viewing. (DOCX 397 kb) [file 12966_2018_735_MOESM1_ESM.docx]
